# *Staphylococcus intermedius* infection with splenic abscesses in a patient with acute lymphoblastic leukemia

**DOI:** 10.1007/s00277-023-05208-3

**Published:** 2023-04-04

**Authors:** Lisa Hauptmann, Danica Midic, Farina Eigendorff, Amer Malouhi, Bernhard Theis, Hermann Kißler, Jürgen Rödel, Florian Prims, Andreas Hochhaus, Sebastian Scholl, Ulf Schnetzke

**Affiliations:** 1 Klinik für Innere Medizin II, Hämatologie und Internistische Onkologie, Universitätsklinikum Jena, Am Klinikum 1, 07747 Jena, Germany; 2 Klinik für Innere Medizin III, Nephrologie, Universitätsklinikum Jena, Jena, Germany; 3 Institut für Diagnostische und Interventionelle Radiologie, Universitätsklinikum Jena, Jena, Germany; 4 Institut für Rechtsmedizin Sektion Pathologie, Universitätsklinikum Jena, Jena, Germany; 5 Klinik für Allgemein-, Viszeral- und Gefäßchirurgie, Universitätsklinikum Jena, Jena, Germany; 6 Institut für Medizinische Mikrobiologie, Universitätsklinikum Jena, Jena, Germany; 7 Klinik für Innere Medizin, Hämatologie und Onkologie, SRH Klinikum Naumburg, Naumburg, Germany

Dear Editor,

We report a case of disseminated *Staphylococcus intermedius* infection causing multiple spleen abscesses with consecutive splenectomy in a patient with acute lymphoblastic leukemia.

**Fig. 1:**
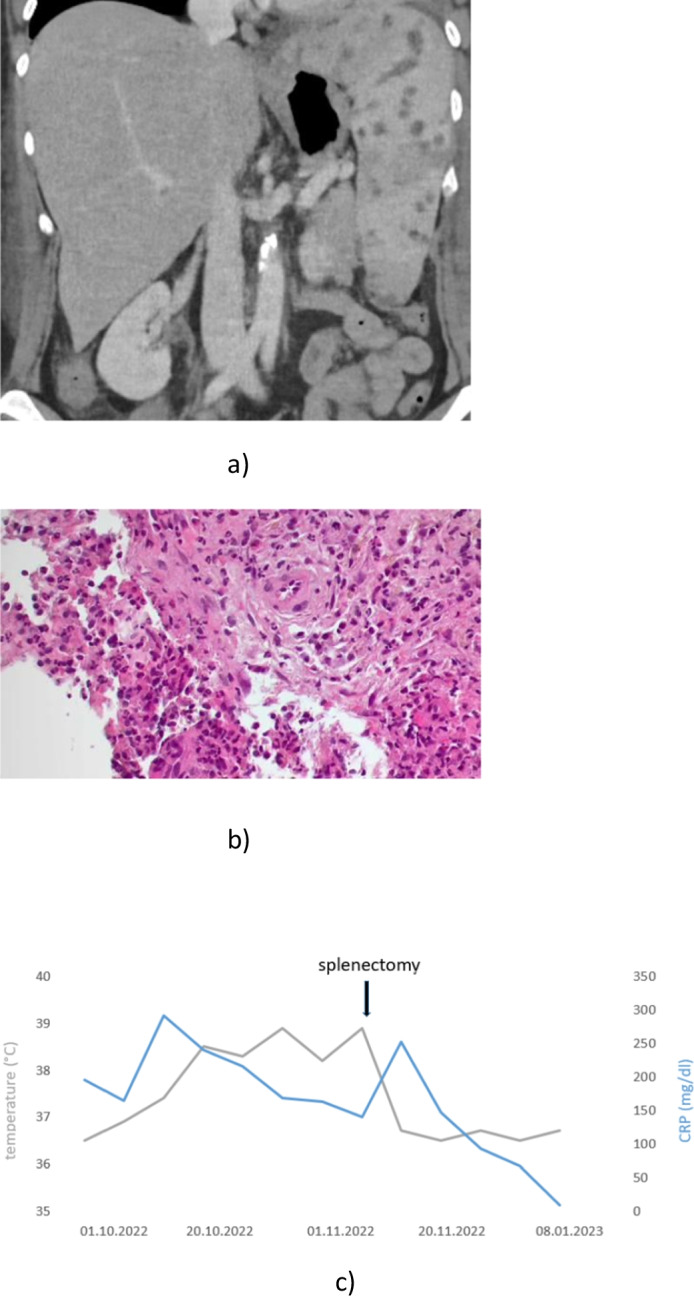
**a** Splenic abscesses on computed tomography scan. **b** Histology of splenic tissue demonstrating neutrophil infiltration caused by the *Staphylococcus intermedius* infection. **c** Body temperature (°C) and C-reactive protein (CRP) before and after splenectomy

A 49-year-old female patient underwent induction chemotherapy in accordance with the Germany Multicentre Study Group (GMALL) 08/2013 protocol for a precursor B-cell acute lymphoblastic leukemia (ALL). Fever episodes occurred during neutropenia, accompanied by intermittent left flank pain and increased inflammation parameters with a maximum C-reactive protein of 368.9 mg/l (normal range, < 5.0 mg/l). Extensive microbiological diagnostics including multiple blood and urine cultures did not reveal any relevant pathogens. Despite broad-spectrum antimicrobial therapy including a combination therapy of meropenem, linezolid, and fosfomycin, later ceftazidime, tigecycline, and liposomal amphotericin B, no improvement of clinical and laboratory parameters was achieved. Abdominal computed tomography scan revealed multilocular splenic abscesses (Fig. 1a). A transthoracic echocardiography showed no evidence of endocarditis and a magnetic resonance imaging (MRI) of the brain did not reveal any cerebral septic abscess. A diagnostic spleen puncture was performed and no causative pathogen or evidence of leukemic infiltration was found. Due to the persistently uncontrollable infection, splenectomy was performed. The histological work-up showed a pronounced infiltration by neutrophilic granulocytes (Fig. 1b). *Staphylococcus intermedius* was identified from splenic abscessus material as the causative pathogen by isolation on Columbia sheep blood agar and identification using MALDI-TOF (Vitek MS, bioMérieux, Nürtingen, Germany). Following splenectomy, febrile episodes and flank pain ceased and a distinct decline in inflammation parameters was recorded (Fig. 1c). With regard to ALL, a sustained complete remission without evidence of measurable residual disease (MRD) was detected by bone marrow puncture. One month after splenectomy, chemotherapy was continued with first consolidation according to the GMALL 08/2013 protocol. During the following neutropenic episodes, the patient received prophylactic therapy with cefazolin in addition to standard antimicrobial prophylaxis. No infectious complications occurred during the following cycles of chemotherapy with ongoing negativity for MRD of the ALL.

Hepatosplenic abscesses in AML patients during neutropenia are often caused by disseminated candidiasis [1]. *Staphylococcus intermedius* rarely occurs in humans and is much more common in animals such as dogs [2]. A review of the literature revealed that twenty-seven of 33 cases (75.8%) had exposure to dogs [3]. The patient described here frequently works with dogs every day due to her occupation. The vast majority of infections cause skin abscesses and wound infections [4–6]. Device-associated infections, brain abscesses, or even meningitis may occur rarely [3, 7, 8]. Rarely, spleen abscesses have been reported due to *Staphylococcus* spp. [9]. All patients reported were immunocompetent. Here, we present a case of splenic abscesses due to a disseminated infection with *Staphylococcus intermedius*. Even though the patient was treated in accordance to antimicrobial resistogram, clinical and laboratory parameters did not improve until splenectomy as a causative treatment option was performed.

When untreated, splenic abscess has a high mortality rate reaching up to 70% but can be reduced by applying appropriate treatment to less than 1%. High-dose parenteral broad-spectrum antibiotics play an important role in the treatment of splenic abscesses. Even though percutaneous aspiration and drainage approaches are becoming more popular, splenectomy remains the gold standard especially in patients with multilocular abscess [10, 11].

Usually, infections due to *Staphylococcus intermedius* are susceptible to glycopeptides (vancomycin), macrolides (erythromycin), aminoglycosides, and most often penicillins [3]. Despite long-term antibiotic treatment, according resistogram the patient described here did not show any clinical and laboratory improvement until splenectomy was performed.

To our knowledge, this is the first report of clinical manifestation of *Staphylococcus intermedius* infection causing splenic abscesses in an immunocompromised patient. In case of persistent signs and symptoms of infection despite broad-spectrum antibiotic treatment, surgical approaches such as percutaneous aspiration or splenectomy should be considered. This case also illustrates that immunocompromised patients require very close clinical observation due to a high rate of morbidity and mortality caused by infectious diseases.
